# Book Reviews

**Published:** 1914-01

**Authors:** 


### BOOK REVIEWS

*Books mentioned may be inspected at and ordered through this office. So far as possible, books received in any month will be reviewed in the issue of the second month following.*

---

EARLY PULMONARY TUBERCULOSIS, Diagnosis, Prognosis and Treatment, John B. Hawes, 2d, M. D., Boston. Published by Wm. Wood & Co., N. Y., 114 pages, illustrated, $1.50.

The author speaks with abundant experience, as an expert and, therefore, modestly and in a very conservative way. For this reason it is difficult to select passages for review as especially novel or arousing debate. The book consists largely of appendixes which should be read as well as the text and, altogether, it is a common-sense, orthodox presentation of professional views, supported with personal familiarity.

---

STAMMERING AND CONATE DEFECTS OF SPEECH. C. S. Bluemel, Boulder, Col., published by G. E. Stechert, N. Y., London, Leipzig and Paris. Two volumes of about 275 pages each, $5.00.

The first volume deals mainly with the psychology of stammering, the second with systematic pronounciation exercises for its treatment. Aphasia and other conditions are included and allusion is made to organic nervous conditions. Defects of speech are a very serious handicap both socially and economically. We recall a pitiable case of an exceedingly beautiful and otherwise attractive girl who could scarcely express her thoughts except in music. Employed in an institution for the feeble minded, she was, even here, largely excluded from the social life of the numerous staff. While fluency of expression is a somewhat dubious asset, marked incapacity for verbal communication is still more unfortunate. The persistance of speech defects and the comparative rarity with which the physician is consulted about them, indicate that there is need of instruction in this branch.

BUREAU OF ETHNOLOGY, Bulletin 53, CHIPPEWA MUSIC II., by Frances Denmore. This supplements Bulletin 45.

These volumes contain many songs with notes and words, the latter translated also, illustrations of the things, and customs to which the songs apply, and a critical study of the tonality, tempo, rhythm, &c, of Chippewa music.

---

THE UNEXPURGATED CASE AGAINST WOMAN SUFFRAGE, Sir Almoth E. Wright, M. D., F. R. S., published by Paul B. Hoeber, 69 E. 59, N. Y., 188 pages, $1.00.

While the word "unexpurgated" did not suggest to us the sense in which it might apply to Ovid or Byron, we expected to find a militancy of expression that might lead to arson of Sir Almoth's residence or office or to personal violence at the hands of the suffragettes. On the contrary, we find a logical and temperate presentation of the arguments against suffrage, objectionable only as any argument as to fitness must seem so to one to whom fitness for anything is denied. While the work is logical, Sir Almoth's opponents can readily find, indeed have already reiterated, the arguments in rebuttal. It must be understood that, in England, the case of woman suffrage is far different to what it is in the States, including physical means as well as purely intellectual discussions. This is excused on the ground that the Englishman is not amenable to argument and moral suasion. Whether there is this marked radical difference or whether the Englishmen in the States, still amounting to about 25 per cent. of the population, have been radically changed by environment, we doubt. But granting this excuse, it is the best reason why violent measures will fail. Meantime, peaceable measures have demonstrated that, in this country and probably others, women will get the suffrage as soon as a majority actually want it and have actually taken their places among men as equal producers of wealth and moulders of public opinion.

---

PRACTICAL MEDICINE SERIES, under general editorial charge of Charles L. Mix, A. M., M. D.; Vol. 7, OBSTETRICS, edited by Joseph B. De Lee, A. M., M. D. and Herbert M. Stone, M. D. The Year Book Publishers, Chicago, $1.35, price for the annual series of ten volumes, $10.00.

As previously stated, this series is the most practical and thorough review of periodic literature now published. We note especially in this volume a very thorough review of the Aberhalden test for pregnancy and a discussion in simple terms of the principles involved and their potential extension to other problems in diagnosis.

The Medical Record Visiting List for 1914, Wm. Wood & Co., N. Y. The regular lists are prepared for 30, 60 and 90 patients a week, at $1.25, $1.50 and $2.00 respectively. Special forms with calf skin wallet for 30, and 60 patients, with replaceable books for six months, are sold at prices ranging from $2.50 to $4.00, carriage prepaid.

The Practitioner's Visiting List for 1914. An invaluable pocket-sized book containing memoranda and data important for every physician, and ruled blanks for recording every detail of practice. The Weekly, Monthly and 30-Patient Perpetual contains 32 pages of data and 160 pages of classified blanks. The 60-Patient Perpetual consists of 256 pages of blanks alone. Each one in wallet-shaped book, bound in flexible leather, with flap and pocket, pencil with rubber, and calendar for two years. Price by mail, postpaid, to any address, $1.25. Thumb-letter index, 25 cents extra. Descriptive circular showing the several styles sent on request. Lea & Febiger, Publishers, Philadelphia and New York.

Both of these lists are in established use, contain dose tables, guides for emergent cases and other useful information. We are at a loss to express a preference between them, even if it were fair to do so. One of the publishers remarks with a sarcasm justified by the dilatory reviews sometimes made, that a review next July will be of no value. We regret that neither list was received till the December issue was in press.

---

Transaction of the American Urologic Association, 12th Annual Meeting at Boston, April 15-17, 1913. Printed by the Riverdale Press, Brookline, under the direction of the Publication Committee, Hugh Cabot, Richard Frothingham O'Neil and George Gilbert Smith.

Our territory is represented by Drs. James A. Gardner and Burton T. Simpson, in a paper on the Relation to Multiple Adenomata to the Etiology of Enlargement of the Prostate. The various monographs included in the transactions are of a high order and show the advances being made in this specialty.

---

Glycosuria and Diabetes, Dr. F. M. Allen. This book, previously reviewed and originally published by W. M. Leonard, has been taken over by the Harvard University Press of Cambridge, Mass.

Causes and Cures of Crime. Thomas Speed Mosby, Jefferson City, Mo., published by the C. V. Mosby Co., St. Louis, 354 pages, illustrated, $2.00.

The author is a lawyer, formerly Pardon Attorney of the State, and has already published works on Capital Punishment, Youthful Criminals, Alcoholism and Crime, Mothers of Bad Boys and other essays. While prepared to discuss the subject from its legal aspects and to avoid the errors of fact and anticipate obstacles to theoretic reform which hamper one without training in the law, the author is not content to discuss Crime as at present punished but treats the subject broadly, from the sociologic and psychologic standpoint. Many of the illustrations represent instruments for scientific gauging of nervous and mental status—collated in a chapter on the New Penology. Indeterminate Sentence and Parole is awarded a lengthy chapter with a list of provisions actually established in various states. The value of finger print identification is illustrated by three photographs of persons closely resembling one another but showing marked differences in the finger prints. The irony of "Twentieth Century Civilization," is illustrated by a photograph of two prisoners in the stocks and a third at the whipping post although personally, we regard the whipping post as, at least, a debatable institution. Taken as a whole, the work is scholarly, hopeful, benevolent and wise.

---

Pyorrhoea Alveolaris, Fredrich Hecker, B. S., D. D. S., A. M., M. D,, Kansas City, published by the C. V. Mosby Co., St. Louis, 157 pages, illustrated, $2.00.

Nine bacteria are most commonly found in this condition. the staphylococcus pyogenes, albus, aureus, citreus, and fetidis, the streptococcus pyogenes, the bacillus pyocyaneus, diplococcus pneumonia, the leptothrix buccalis and the spirochete refringens. These are discussed in detail. Autogenic vaccines are regarded favorably. The clinical manifestations of the disease, and the various diagnostic and therapeutic methods are fully discussed.

---

Medical Research and Education. The Science Press, New York and Garrison, N. Y., 536 pages.

This is the second volume in a series on Science and Education, edited by J. McKeen Cattell but of independent scope and appealing especially to medical interest. It contains a number of essays by distinguished laboratory workers and educators and, while not directly dealing with technical achievements, is of great value as throwing light on the history and general ethics and potentialities of research. It is by no means a narrow plea for vivisection but has a broad scope.

---

Case Histories in Paediatrics. John Lovett Morse, A. M., M. D., Boston; published by W. M. Leonard, Boston; 638 pages, illustrated; $3.00.

This is the second edition, indicating a professional appreciation of an excellent work. While the clinical case method is followed, general methods of diagnosis, &c. are systematically presented at the outset and the cases are logically grouped.

SURGICAL EXPERIENCES IN SOUTH AFRICA. Being mainly a clinical study of the nature and effects of injuries produced by bullets of small calibre, George Henry Makins, C. B., F. R. C. S., London. Published by Henry Frowde and Hodder and Stoughton, represented by Oxford University Press, American Branch, 35 W. 32, N. Y., 504 pages, $3.75.

It is rather to be regretted that this book has appeared so long after the Boer War of 1899-1900, when the author made the observations as consulting surgeon to the South African Field Force of the British Army. It is however, a second edition and while surgery has made steady advances, no new discoveries nor changes of methods of military surgery have occurred such as to relegate this work to the class of "historically interesting." Both in its strictly scientific features, covering a wide range of surgery, through especially military traumatism and in its adaptations to the training of military surgeons, it is still modern aud authoratative. Very little attention is given to executive details and, in our opinion, the usefulness of the book, especially for foreign readers and for future campaigns, is enhanced by this fact.

Some interesting comparisons of casualties in different wars are given. For instance, the Kimberly Relief Force (English) had 2 per cent. of its troops killed and 9.6 per cent. wounded, whereas at Waterloo, the percentages were 4.85 and 16.25; for the Crimea, 2.81 and 12.35; for the Franco-Prussian War (German) 1.97 and 10.83, and for the Russo-Turkish War (Russian) 10.92 and 23.75. As the author points out, the Boer policy was to kill without being killed and while the losses were relatively less than those of the English, the method of warfare necessarily tended to reduce the losses of the English also.

---

TRANSACTIONS OF THE AMERICAN ASSOCIATION OF OBSTETRICIANS AND GYNAECOLOGISTS, Vol. 25, for 1912. Published by the Maple Press of York, under the direction of the Secretary, Dr. E. Gustav Zinke of Cincinnati.

A convenient list of members, with brief biographic data, is given. Without venturing to select any of the numerous scientific papers for review, it is significant that those dealing with strictly obstetric subjects are comparatively few and that even in the surgical papers, a tendency to discuss complications and even conditions not directly involving the sexual organs, is manifest. We mention this in no spirit of censure; it merely shows the development along new lines of discovery and thought and the broadness of view of modern specialism. In our own experience, we note the same tendency to diversion from specialism in the narrow, simple form, to more recondite borderland cases and more particularly in those cases in which the question as to the legitimacy of including such cases in a given specialty is decided by other physicians. The paucity of papers dealing with obstetrics not involving major surgery, seems to confirm the impression derived from a contrast between modern and quite old books on obstetrics—that this branch of medicine is nearer the limit of human perfection than any other. We wish that every one could read this masterly collection of monographs and we feel that the published book reflects great credit on the Secretary.

---

The Principles and Practice of Medical Hydrology. R. Fortescue Fox, M. D., F. R. Met. Soc., London. University of London Press, represented by the Oxford University Press, American Branch, 35 W. 32d, N. Y.; 295 pages; $2.00.

The physiology of bathing, including observations on the functions of the skin, bodily heat, etc., general principles of hydrotherapy and reference lists of European spas and springs are given. A considerable but not comprehensive list of special diseases to which hydrologic treatment is applicable is given and details are well discussed. The fact that the author does not discuss hydrotherapeutic measures for every conceivable disease, affords a sense of confidence. Throughout the work, we find more space devoted to balneology and greater emphasis placed on the external than on the internal use of mineral waters. This fact also inspires confidence.

---

Dysenteries, Their Differentiation and Treatment. Leonard Rogers, M. D., F. R. C. P., B. S., etc., Calcutta. Published by Henry Frowde and Hodder & Stoughton London. Represented by Oxford University Press, American Branch, 336 pages, $3.75.

A few beautiful colored illustrations are included in this work, quite equal to American standards. The first chapter gives the history of dysentery up to the differentiations of the various types. Amoebic and Bacillary Dysenteries are sharply distinguished as are the pathogenic from the harmless amoebae. "Other forms of dysentery," including the effects of balantidiun, bilharzia, tricomonas and intestinal worm, Hill Diarrhoea and sprue are well discussed and the author regards it as probable that nonspecific colitis may resemble dysentery. The multiplicity of forms of dysentery is well illustrated by the table of sugar reactions of seven types of bacilli. While dysentery is not a common disease in this climate, it was frequently mentioned by the older practitioners and it is quite likely that mildness of infection may have misled the present day profession as to its frequence and importance. At any rate, dysentery is—or rather dysenteries are—prevalent in many parts of the world and specific infections are liable to be introduced elsewhere by travel and commerce. Hence a work of this nature and scope, is a welcome addition to the library and one which should be widely read.

---

Observation on Pregnancy and Labor and on the Maladies of Woman and the New-born. Francois Mauriceau, Master of Arts and Ex-prevost of the company of Sworn Master Surgeons of the City of Paris, published at Paris by the Company of Associated Libraries, 1738.

These observations consist of a series of 700 case reports and a supplement of 150 final observations, covering altogether the years from 1669 to 1704. The first series is indexed. This method of medical writing, recently revived, has the disadvantage of lack of systematic arrangement but the advantage that, however much theories and knowledge may change, the observations themselves are, for the most part, always available as records of facts in recognizable form.

In reading an old medical book, one is surprised to find how much of what he supposes to be comparatively modern, is really old. Numerous observations cover the abnormal presentations, abortions, prolapse of the funis, placenta previa, premature delivery, etc., in fact the index is surprisingly modern in its extent, except for certain peculiarities of nomenclature. We select, therefore a few cases of peculiar interest or showing notable differences from or similarity to modern obstetrics.

Too frequent applications of butter during labor are condemned and death of the child is attributed to the use of too cold butter.

In a case in which the author has terminated expulsive efforts of two days' duration by extracting a "false germ" of the size of a small hen's egg from the uterus, the nurse showed him another membranous mass passed the day previously and which she and the attending physician had regarded as another false germ. After careful inspection, the author convinced them that this was a section of chicken gut used over the tip of a rectal syringe to facilitate introduction, the patient having piles.

Conception without rupture of hymen, without previous menstruation and occurring during menstruation. The last case was of a woman aged 44, who had had intercourse for eleven or twelve years only during menstruation, believing that she could not conceive at that time. After realizing that she had conceived, fears were entertained that the child would be abnormal but it was perfectly healthy. The author mentions voluntary sterility in several instances, but, so far as we have noted, only from continence. Various other well known causes of sterility are mentioned, including imperforate hymen (one case rendered fertile by operation) tapir-like cervix (one case of sterility by first husband, fertility by second), obesity, &c.

Gonorrhoea is rather frequently mentioned but is apparently not always sharply distinguished from other discharges. However, the infection of wives from husbands is well recognized and three cases are reported in little girls, in one on account of precocious sexuality—the author calls her a little minx or words to that effect in French—in two others, aged respectively six and seven who had been seduced or raped by servants, without penetration. In the latter case, he observed that the villians ought to have been burned alive. Gonorrhoea does not seem to have been observed as a cause of blindness nor definitely recognized as a cause of sterility.

That eugenics is not entirely a new thought is evidenced by the selection of six cases of healthy infants born of infirm mothers, to illustrate exceptions to a rule.

Complete procidentia is mentioned several times, both in parous women and in two nulliparae who had sustained the prolapse at age of 16 and 22, respectively, from muscular strain. Successful reposition was accomplished in each case after rest, purgation and bleeding and pessaries were introduced.

A case indexed as vomiting of meconium, is described as continued vomiting of a new born infant, the vomiting ultimately becoming dark and the diagnosis being in doubt between meconium, bile and blood. Today, the last tentative diagnosis seems most probable. The child recovered.

Under the title of venereal disease of pregnant women, syphilis alone is meant, except in one case described as virulent gonorrhoea due to the husband and leading to a formation of bubo. In four cases, delivery of a normal infant and cure of the mother is reported, under the use of mercury to salivation. No unsuccessful cases are reported, although undoubtedly some of the numerous cases of abortion, false germ, &c, were of syphilitic origin. Perhaps we may well act on the hint to use heroic doses of mercury in pregnant women.

The author repeatedly emphasizes our inability to determine the sex of the unborn child—an opinion of interest both with relation to old-time notions and modern attempts to the contrary. He also reports triumphantly, a case in which the husband had visited an astrologer and had returned with his head filled with what he had predicted from the inspection of the stars to show that the exact date of parturition could not be foretold.

One case of superfoetation is mentioned, the woman giving birth first to a small foetus, decomposed but surrounded by its sac and then to an infant of six and one-half months, preceded by discharge of amnittic fluid, alive but not surviving. From various circumstances, he concluded that the dead foetus antedated the second pregnancy. Superfecundation is not explicitly mentioned.

Six cases of post mortem Caesarian section are mentioned.

Malaria treated by cinchona, dysentery and a great variety of diseases occurring during pregnancy, are described. Some of the most interesting allusions are to small pox. For example, he mentions a case of a woman who contracted variola in the second month of pregnancy, recovered and was delivered at six and one-half months of a foetus, apparently recently dead, presenting more than twenty typic pustules. On the other hand he reports a case and mentions having had a number of others in which small pox neither interrupted pregnancy nor was transmitted to the foetus. He also mentions small pox in the puerperium.

Note: Owners of ancient medical books are invited to contribute abstracts of them.

